# Dopaminergic REST/NRSF is protective against manganese-induced neurotoxicity in mice

**DOI:** 10.1016/j.jbc.2024.107707

**Published:** 2024-08-22

**Authors:** Edward Pajarillo, Sanghoon Kim, Alexis Digman, Itunu Ajayi, Ivan Nyarko-Danquah, Deok-Soo Son, Michael Aschner, Eunsook Lee

**Affiliations:** 1 Department of Pharmaceutical Science, College of Pharmacy and Pharmaceutical Sciences, Florida A&M University, Tallahassee, Florida, USA; 2 Department of Biochemistry, Cancer Biology, Neuroscience and Pharmacology, Meharry Medical College, Nashville, Tennessee, USA; 3 Department of Molecular Pharmacology, Albert Einstein College of Medicine, Bronx, New York, New York, USA

**Keywords:** mitochondrial fission/fusion, apoptosis, dopaminergic neurons, manganese, mitophagy, NRSF, REST, oxidative stress

## Abstract

Chronic exposure to elevated levels of manganese (Mn) may cause a neurological disorder referred to as manganism. The transcription factor REST is dysregulated in several neurodegenerative diseases, such as Alzheimer’s disease and Parkinson’s disease. REST upregulated tyrosine hydroxylase and induced protection against Mn toxicity in neuronal cultures. In the present study, we investigated if dopaminergic REST plays a critical role in protecting against Mn-induced toxicity *in vivo* using dopaminergic REST conditional knockout (REST-cKO) mice and REST loxP mice as wild-type (WT) controls. Restoration of REST in the substantia nigra (SN) with neuronal REST AAV vector infusion was performed to further support the role of REST in Mn toxicity. Mice were exposed to Mn (330 μg, intranasal, daily for 3 weeks), followed by behavioral tests and molecular biology experiments. Results showed that Mn decreased REST mRNA/protein levels in the SN-containing midbrain, as well as locomotor activity and motor coordination in WT mice, which were further decreased in REST-cKO mice. Mn-induced mitochondrial insults, such as impairment of fission/fusion and mitophagy, apoptosis, and oxidative stress, in the midbrain of WT mice were more pronounced in REST-cKO mice. However, REST restoration in the SN of REST-cKO mice attenuated Mn-induced neurotoxicity. REST’s molecular target for its protection is unclear, but REST attenuated Mn-induced mitochondrial dysregulation, indicating that it is a primary intracellular target for both Mn and REST. These novel findings suggest that dopaminergic REST in the nigrostriatal pathway is critical in protecting against Mn toxicity, underscoring REST as a potential therapeutic target for treating manganism.

Chronic exposure to elevated levels of manganese (Mn) *via* occupational settings, such as mining and welding industries, and environmental settings, such as exposure to the pesticide, maneb, and Mn-contaminated drinking water, may lead to its accumulation in the basal ganglia of the brain and cause a neurological disorder referred to as manganism (1, 2, 3, 4). Mn accumulation and its selective toxicity attribute to the high levels of the expression of the divalent metal transporter 1 in the basal ganglia, such as the striatum, pallidum, and substantia nigra (5). Moreover, the dopamine transporter (DAT) expressed in the dopaminergic neurons binds Mn and deposits it in dopamine-rich regions, as supported by the study that DAT knockout (KO) mice led to decreased Mn accumulation in the striatum compared to the wild-type (WT) mice (6), resulting in dopaminergic neurons being more vulnerable (7).

The pathological signs and symptoms of Mn toxicity, referred to as manganism, resemble those inherent to Parkinson’s disease (PD), exhibiting impairment of extrapyramidal motor function as well as cognitive and emotional deficits in humans (8, 9) and experimental animal models (10, 11). These Mn-induced behavioral deficits are closely associated with dopaminergic neuronal dysfunctions in the nigrostriatal pathway (12, 13, 14, 15). Furthermore, Mn is considered a risk factor in the development of PD, contributing to the onset and progression of idiopathic PD (for a comprehensive review, (16)). Studies in mice have also shown that Mn exacerbated toxicity in LRRK2 G2019S, the most common genetic mutation linked to familial and sporadic PD, compared to WT (17).

It is well established that Mn dysregulates dopaminergic functions, such as reducing dopaminergic neurotransmission and inducing neuroinflammation (18, 19, 20). Mn decreased the expression of tyrosine hydroxylase (TH), a rate-limiting enzyme for dopamine synthesis, and inhibited TH phosphorylation (18, 21, 22, 23). However, the molecular mechanisms underlying Mn-induced dopaminergic toxicity are yet to be fully understood. At the cellular level, Mn preferentially accumulates and targets mitochondria (24), causing mitochondrial toxicities, such as impairment of mitochondrial membrane potential, ATP depletion, imbalance of fission-fusion dynamics, oxidative stress, and apoptosis (10, 17, 25, 26, 27, 28, 29). Recently, Mn-induced autophagy impairment has been drawing significant attention. Mn disrupts the autophagy process by increasing the earlier step of autophagosome formation but inhibiting autophagosome-lysosome fusion steps, possibly due to lysosomal defects, leading to cell death in PC12 cells (28) and thus preventing autophagic clearance of damaged proteins (30). Oxidative and nitrosative stress are posited to be responsible for Mn-induced autophagy dysregulation in mice and neuronal cells (28, 31). Mn-induced impairment of autophagy is closely correlated with dopaminergic toxicity and neurodegeneration in rats (32). Moreover, Mn-induced lysosomal defects cause lysosomal leakage of cathepsin B, which leads to inflammasome activation in microglial cells and subsequent exosomal propagation of inflammasomes into the adjacent neurons, resulting in neuronal damage (33). Mn also impairs mitophagy, a selective autophagy that degrades old and damaged mitochondria. Although mitophagy initiates with mitochondrial components, it undergoes most of the autophagy process, working with autophagy proteins, including p62, LC3, and beclin1 (34). Impairment of mitophagy can lead to energy deficiency, oxidative stress, inflammation, and apoptosis (35, 36, 37), further highlighting mitophagy dysfunction in Mn toxicity.

Repressor element 1-silencing transcription factor (REST), a transcription factor (TF), also known as neuron-restrictive silencer factor, has been shown to exert neuroprotection in several animal models of neurodegenerative diseases, including PD and Alzheimer’s disease (AD) (38, 39). Higher levels of REST are correlated with better cognitive function in the normal aging brain, but lower levels and abnormal cellular localization of REST are observed in AD and PD patients (40, 41). Interestingly, REST deficiency leads to impaired autophagy and consequent neurotoxicity (42). Nonetheless, the mechanisms involved in REST-induced neuroprotection remain to be fully understood. In the normal aging human brain, REST is induced and regulates a network of genes that mediate cell death and stress resistance (40). REST also induces neuroprotection by repressing genes involved in oxidative stress and cell death while upregulating genes that promote neuronal survival in experimental models (40, 43). Growing evidence suggests that REST is dysregulated in Mn toxicity. Our previous studies revealed that Mn decreased REST transcription, while REST overexpression attenuated Mn toxicity in neuronal cultures, further supporting the role of REST in neuroprotection (18). In addition to neuronal cells, REST increased the transcription of astrocytic excitatory amino acid transporter 2 and attenuated Mn-induced decrease in excitatory amino acid transporter 2 expression, protecting against Mn-induced excitotoxicity in astrocyte-neuron cocultures (44). These findings suggest that REST induces protection *via* several mechanisms in different cell types and environments, providing immense potential for treating neurodegenerative diseases, including Mn-induced neurotoxicity, PD, and AD. Although neuronal REST has been shown to be protective in animal models of AD and PD (39, 40), its role in dopaminergic neurons, particularly in relation to Mn-induced neurotoxicity *in vivo,* has yet to be investigated. Therefore, we deleted dopaminergic REST using conditional knockout (REST-cKO) mice and restored REST using REST-expressing adeno-associated viral vectors (AAVs) in dopaminergic neurons in the nigrostriatal regions of REST-cKO mice to determine the role of dopaminergic REST in Mn-induced neurotoxicity. AAV, a non-enveloped virus engineered to deliver DNA to target cells, has been widely used to express specific proteins in the target area of the brain, providing novel therapeutic approaches and disease models (45). Among several serotypes of AAVs, AAV9 is highly efficient for transgene expression in neurons when directly injected into the brain (46). The infusion of AAV9 particles encoding target genes into the substantia nigra would induce the expression of target proteins like REST in nigrostriatal regions due to the localization of dopaminergic cell bodies in the substantia nigra, innervating the nigrostriatal dopaminergic pathway (45).

In the present study, we investigated the role of dopaminergic REST and its protective effects against Mn-induced neurotoxicity using dopaminergic REST-cKO mice and neuronal cultures. Our findings revealed that the deletion of dopaminergic REST exacerbated Mn-induced neurotoxicity, including motor deficits and cellular toxicities, such as apoptosis, oxidative stress, and impairment of mitophagy, which were restored by REST overexpression in the substantia nigra of REST-cKO mice as well as in neuronal cultures.

## Results

### Dopaminergic REST was selectively deleted in mice by the Cre-lox technology

**Figure 1:**
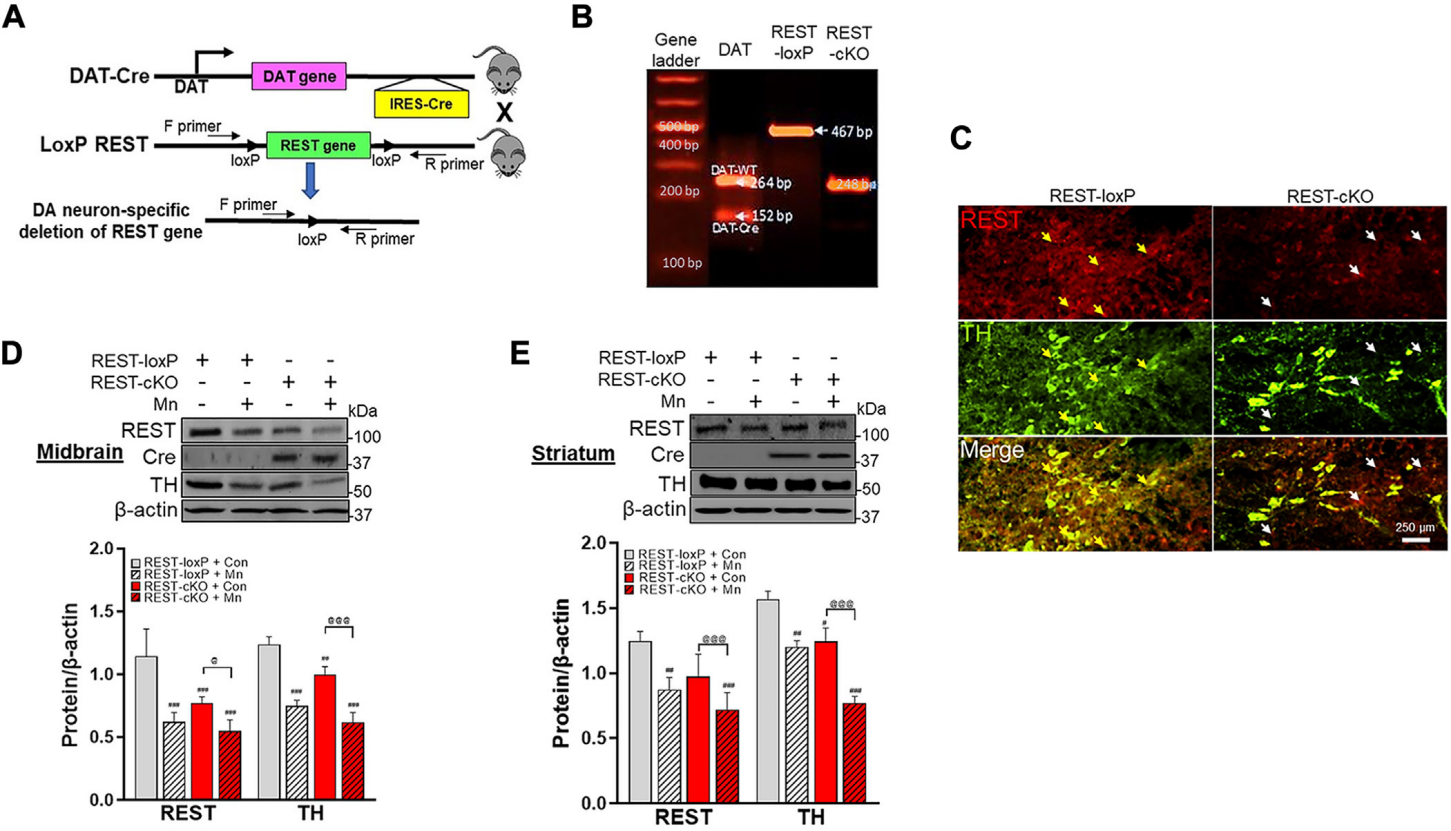
**Validation of REST deletion in dopaminergic neurons and Mn’s effects in REST and TH expression in the substantia nigra and striatum of mice.***A*, deletion of dopaminergic REST was achieved by crossing DAT^IRES.*Cre*^ mice with REST-loxP mice using the Cre-lox strategy. *B*, confirmation of the genomic presence of DAT^IRES.*Cre*^ and loxP sequences in REST-cKO mice. *C*, coronal sections were subjected to immunostaining with antibodies targeting REST and TH, a marker of dopaminergic neurons (*red*). *Arrows* indicate that *yellow* is for colocalization of REST and TH; *white* is for REST only. *D*-*E*, REST, Cre recombinase, and TH proteins were measured in the midbrain (*D*) and striatum (*E*). β-actin was used as a loading control. ^#^*p* < 0.05, ^##^*p* < 0.01, ^###^*p* < 0.001, compared with the controls; ^@^*p* < 0.05, ^@@@^*p* < 0.001, compared with each other (two-way ANOVA followed by Tukey’s *post hoc* test; n = 3). Data are expressed as mean ± SD.

To investigate the potential role of dopaminergic REST in Mn-induced neurotoxicity, we used dopaminergic neuron-specific REST knockout (REST-cKO) mice by crossing REST-loxP mice with DAT-Cre (DAT^IRES.*Cre*^) mice as described in the Experimental procedures and shown in Figure 1*A* (47). Dopaminergic REST-cKO mice express Cre recombinase under the control of the DAT promoter in dopaminergic neurons (48). Genotyping and immunohistochemistry (IHC) data for REST-cKO confirmed the deletion of REST expression in dopaminergic neurons in the midbrain (Fig. 1, *B* and *C*).

### Mn decreased REST mRNA and protein levels in the nigrostriatal regions of the mouse brain

REST^*fl/fl*^ mice were used as the WT control for the dopaminergic REST-cKO mice as REST loxP mice, and the genetic background C57BL/6J mice did not show any difference in behavioral and endogenous REST expression (data not shown). Mice were randomly divided into four groups (two WT and two cKO groups), and one group of mice from each genotype was exposed to Mn by intranasal instillation (Mn 330 μg, administered as manganese chloride (MnCl_2_) 30 mg/kg, daily) for 3 weeks, and the rest of mice from each genotype received water as a vehicle. Twenty-four hours after the last Mn exposure, several behavioral tests were carried out before the endpoint experiments. Results revealed that Cre recombinase was expressed in the midbrain of REST-cKO mice (Fig. 1*D*), accompanied with lower levels of REST protein in the same regions, compared to the REST-loxP/WT control mice (Fig. 1*D*). This lower REST levels in the midbrain of REST-cKO mice may be due to selective REST deletion only in dopaminergic neurons. We also tested if Mn decreases the expression of REST and TH in dopaminergic neurons of WT mice, as previously reported (18). Results showed that Mn decreased TH and REST protein levels in the midbrain of WT mice, with further decreases in REST-cKO mice (Fig. 1*D*) by two-way ANOVA with two independent variables, genotypes, and Mn treatment. Similar trends of protein expression levels of REST, Cre, and TH were also observed in the striatum in which dopaminergic axons are innervated (Fig. 1*E*).

### Dopaminergic REST deletion exacerbates Mn-induced behavioral deficits in mice

**Figure 2:**
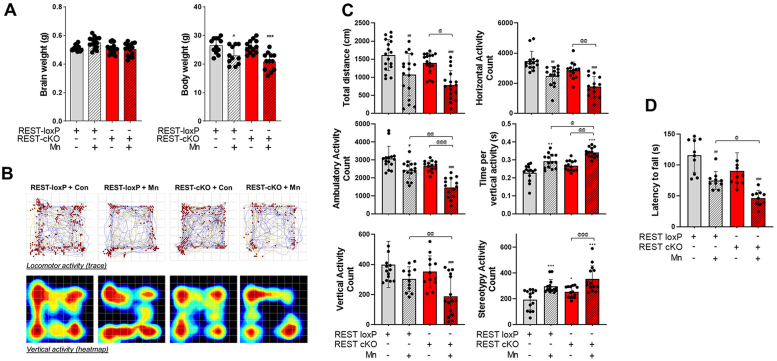
**Deletion of dopaminergic REST exacerbates Mn-induced deficits in movement and motor coordination.***A*, following Mn exposure (Mn 330 μg, administered as MnCl_2_, 30 mg/kg, intranasal instillation, daily for 3 weeks) as described in the Experimental procedures; body and brain weights of mice were measured. *B*-*D*, locomotor activity and motor coordination were assessed as described in the Experimental procedures. *B*, locomotor activities were depicted by the traces for the movements of 1 mouse, with *red* dots representing vertical activity and heatmap of one mouse. *Blue* and *red* colors indicate low and high activity, respectively. *C*, several parameters of locomotor activities, such as total distance traveled, horizontal activity, ambulatory/walking activity, latency for vertical activity, movement speed, and stereotypy activity count, were measured. *D*, motor coordination was assessed by fall latency as time spent on the rotating rod. ^∗∗^*p* < 0.01, ^∗∗∗^*p* < 0.001, ^#^*p* < 0.05, ^##^*p* < 0.01, ^###^*p* < 0.001, compared with the controls; ^@^*p* < 0.05, ^@@^*p* < 0.01, ^@@@^*p* < 0.001, compared with each other (two-way ANOVA with two independent variables of genotypes and Mn treatment, followed by Tukey’s *post hoc* test; n = 15–20). Data are expressed as mean ± SD.

To determine the potential role of dopaminergic REST in Mn-induced behavioral deficits in mice, open-field and rotarod tests were conducted to assess locomotor activity and motor coordination, respectively. Mn is well known to impair movement and motor coordination in rodents (10, 11, 17, 19, 20, 27, 49), which were also confirmed by our results (Fig. 2). In addition, the weights of whole bodies and brains of both WT and REST-cKO mice were assessed to determine if dopaminergic REST is involved in the development and growth of mice. Results revealed that there were no significant differences in the body and brain weights between the WT and REST-cKO groups, and Mn also had no effects on either (Fig. 2*A*). The locomotor and behavioral activities of mice were measured in the open-field arena, assessing their movement patterns, frequency, and the location of vertical movements (Fig. 2*B*). Various parameters of locomotor activity, such as total traveled distance, horizontal activity, ambulatory activity, time to perform a vertical activity, and walking velocity, were measured in WT and REST-cKO mice with or without Mn (Fig. 2*C*). Dopaminergic REST deletion did not alter these parameters compared to the WT mice, except for an increased stereotypy activity in REST-cKO mice compared to WT mice. Motor coordination was also not significantly affected by dopaminergic REST deletion (Fig. 2*D*). On the other hand, Mn further worsened the impairment in total distance traveled, horizontal and walking activity, walking speed, and vertical activity (Fig. 2*C*). Mn also further exacerbated the decreased latency time to fall from the rotarod in REST-cKO mice compared to WT mice (Fig. 2*D*), indicating that dopaminergic REST deletion sensitizes Mn effects on behavioral deficits.

### Dopaminergic REST deletion exacerbates Mn-induced apoptotic signaling in the nigrostriatal pathway of the mouse brain

**Figure 3:**
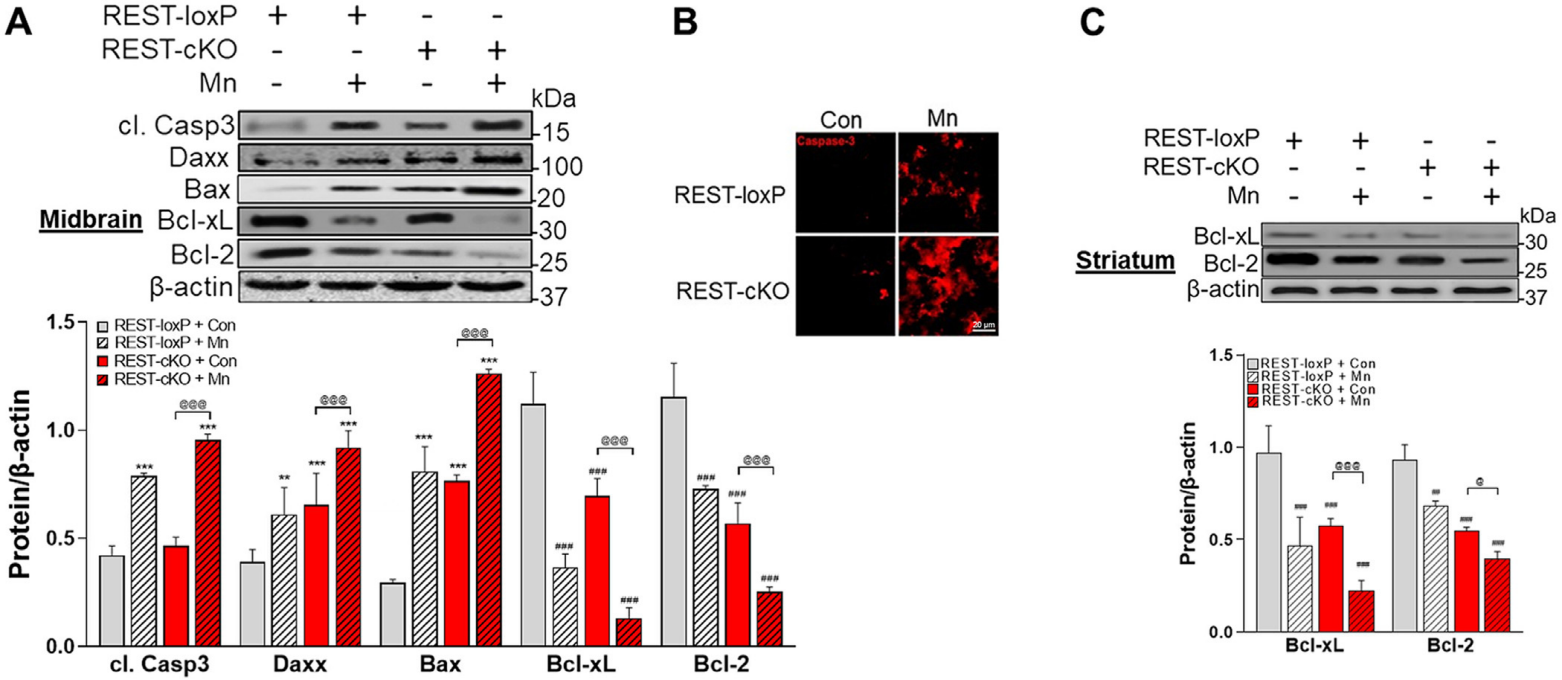
**Deletion of dopaminergic REST exacerbates Mn-induced apoptosis in the midbrain and striatum of mice.***A*, following Mn exposure, protein levels of cleaved caspase-3 (cl. Casp3), Daxx, Bax, Bcl-xL, and Bcl-2 were measured in the midbrain of mice by Western blotting as described in the Experimental procedures. *B*, coronal sections of the midbrain of mice were immuno-stained for caspase-3, a proapoptotic marker. *C*, protein levels of antiapoptotic Bcl-xL and Bcl-2 were measured in the striatum. β-actin was used as a loading control. ^∗∗^*p* < 0.01, ^∗∗∗^*p* < 0.001, ^##^*p* < 0.01, ^###^*p* < 0.001, compared with the controls; ^@^*p* < 0.05, ^@@@^*p* < 0.001, compared with each other (two-way ANOVA with two independent variables of genotypes and Mn treatment, followed by Tukey’s *post hoc* test; n = 3). Data are expressed as mean ± SD.

Mn promotes cell death by increasing proapoptotic proteins and decreasing antiapoptotic proteins in mice (17, 49). To determine if dopaminergic REST deletion would contribute to Mn toxicity and if so, to understand its potential underlying mechanisms, we assessed the Mn-induced apoptotic pathway in dopaminergic neurons. Mn increased levels of proapoptotic proteins such as cleaved caspase-3, Daxx, and Bax, while it decreased antiapoptotic proteins such as Bcl-xL and Bcl-2 in the midbrain of WT mice, which were further exacerbated in REST-cKO mice (Fig. 3*A*). IHC also revealed that Mn increased protein expression of proapoptotic caspase-3, a marker of active apoptosis in the substantia nigra of WT mice, which was further exacerbated in REST-cKO mice (Fig. 3*B*). Mn also reduced antiapoptotic proteins such as Bcl-xL and Bcl-2 in the striatum (Fig. 3*C*), indicating that REST regulates antiapoptotic proteins.

### Dopaminergic REST deletion exacerbates Mn-induced oxidative stress in the nigrostriatal pathway of the mouse brain

**Figure 4:**
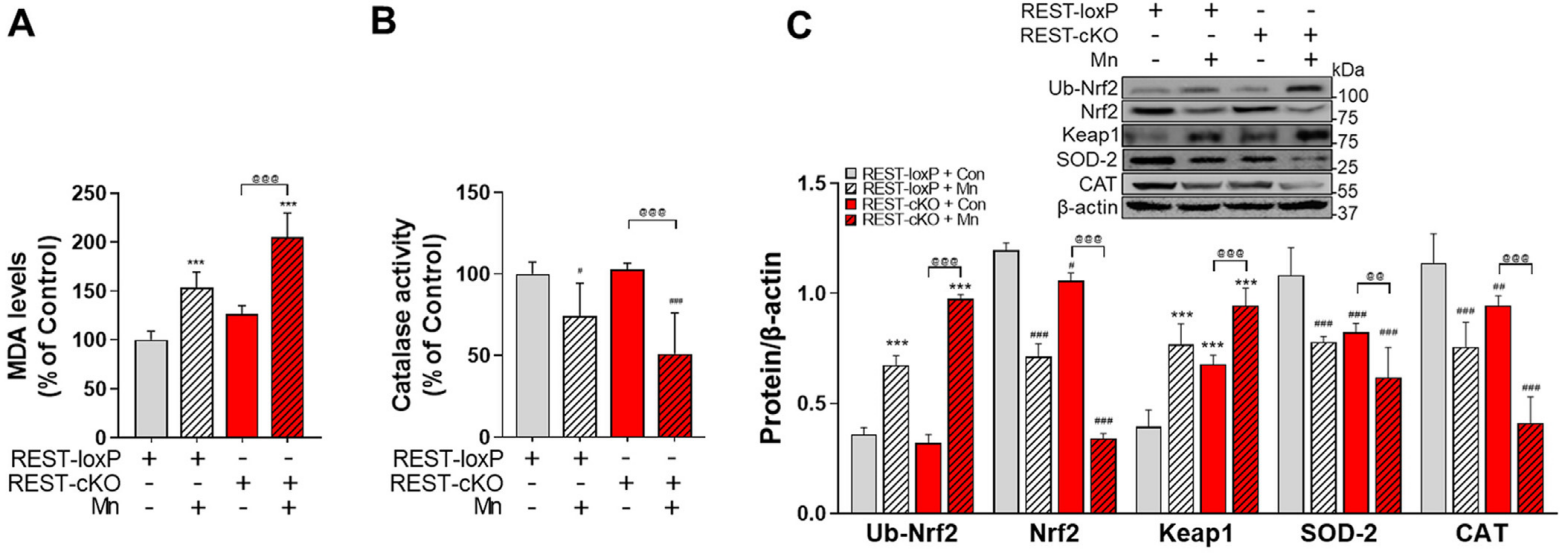
**Deletion of dopaminergic REST exacerbates Mn-induced oxidative stress and impaired expression of antioxidant proteins in the midbrain of mice.***A*-*C*, following Mn exposure, midbrain tissues were assessed for malondialdehyde (MDA) levels, a marker for lipid peroxidation (*A*), catalase activity assay using TBARS assay (*B*), and protein levels of Nrf2, ubiquitinated Nrf2, Keap1, SOD-2, and catalase by Western blotting (*C*), respectively, as described in the Experimental procedures. β-actin was used as a loading control. ^∗∗∗^*p* < 0.001, ^#^*p* < 0.05, ^##^*p* < 0.01, ^###^*p* < 0.001, compared with the controls; ^@@^*p* < 0.01, ^@@@^*p* < 0.001, compared with each other (two-way ANOVA with two independent variables of genotypes and Mn treatment, followed by Tukey’s *post hoc* test; n = 3). Data are expressed as mean ± SD.

As Mn induces oxidative stress and decreases the levels of antioxidant proteins, such as catalase and superoxide dismutase 2 (SOD-2), in dopaminergic neurons (50), we tested if dopaminergic REST deletion causes oxidative stress and exacerbates the Mn-induced oxidative stress in the nigrostriatal regions. We assessed antioxidant catalase activity and malondialdehyde (MDA), a marker of lipid peroxidation, along with protein levels of genes associated with oxidative stress in the midbrain. Results revealed that Mn increased MDA levels in the midbrain of the WT mice, which were further elevated in REST-cKO mice, while basal MDA levels were unchanged in REST-cKO mice compared to the WT mice (Fig. 4*A*). Mn also decreased catalase activity in the midbrain of WT mice, which were further reduced in REST-cKO mice (Fig. 4*B*). Similar results were also observed in SOD-2 protein levels (Fig. 4*C*). REST-cKO mice decreased protein levels of catalase and SOD-2 compared to the WT mice. Moreover, Mn decreased Nrf2 protein levels but increased ubiquitinated Nrf2 and Keap1 protein levels in the midbrain of WT mice, which were further pronounced in REST-cKO mice (Fig. 4*C*). REST-cKO mice also showed higher Keap1 expressions compared to WT mice at both basal and Mn-induced levels (Fig. 4*C*).

### Dopaminergic REST deletion exacerbates Mn-induced impairment of mitochondrial membrane fission/fusion and mitophagy

**Figure 5:**
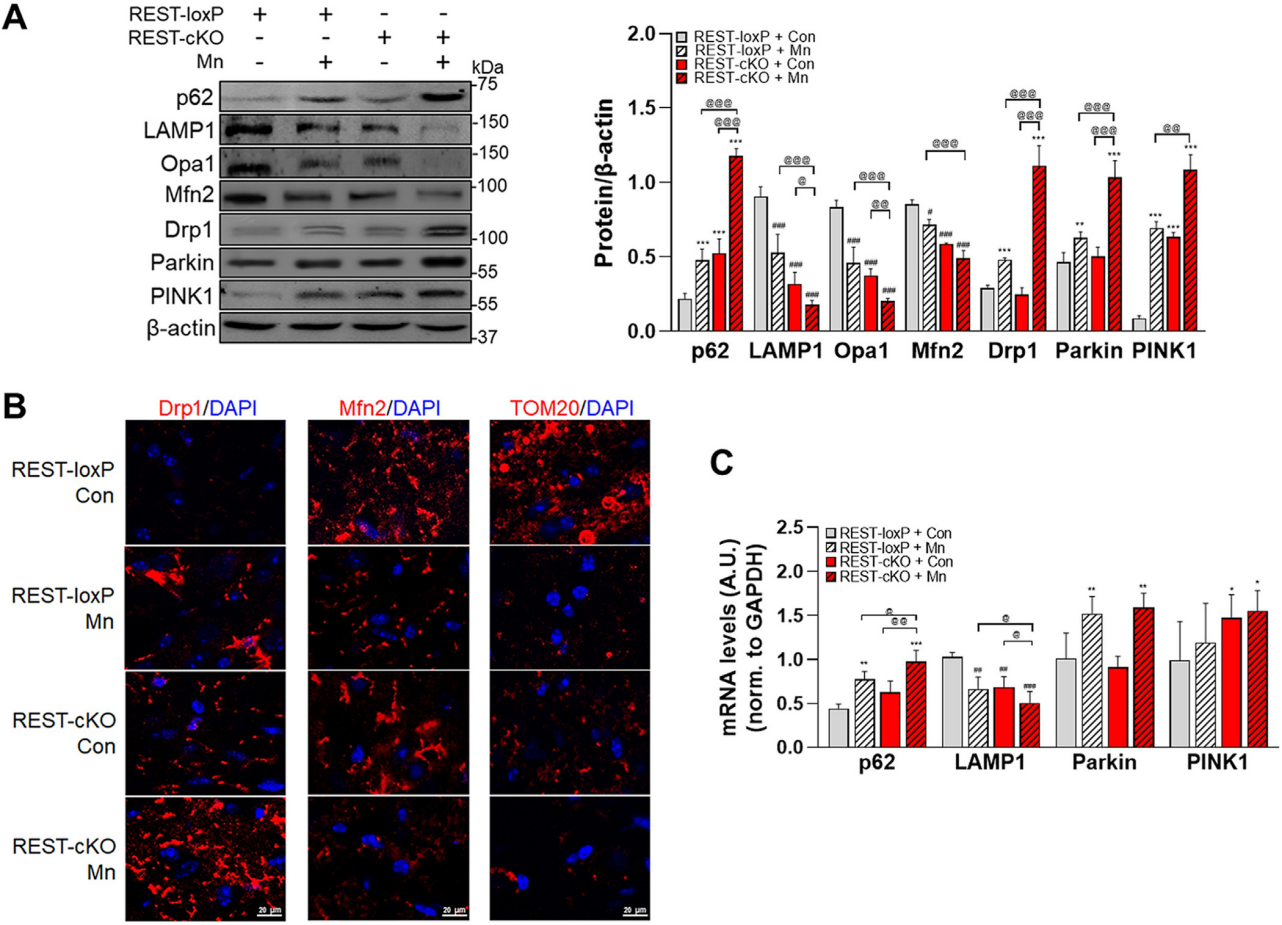
**Deletion of dopaminergic REST exacerbates Mn-induced dysregulation in mitochondrial membrane fission/fusion and mitophagy in the midbrain of mice.***A*, following Mn exposure, midbrain tissues were analyzed for protein of p62, LAMP1, Opa1, Mfn2, Drp1, Parkin, and PINK1. *B*, coronal sections of the mouse substantia nigra region were immunostained for Drp1, Mfn2, and TOM20. *C*, midbrain samples were assessed for mRNA levels of p62, LAMP1, parkin, and PINK1. GAPDH and β-actin were used as loading controls for mRNA and protein, respectively. ^∗^*p* < 0.05, ^∗∗^*p* < 0.01, ^∗∗∗^*p* < 0.001, ^##^*p* < 0.01, ^###^*p* < 0.001, compared with the controls; ^@^*p* < 0.05, ^@@^*p* < 0.01, ^@@@^*p* < 0.001, compared with each other (two-way ANOVA with two independent variables of genotypes and Mn treatment, followed by Tukey’s *post hoc* test; n = 3). Data are expressed as mean ± SD.

Given that Mn impairs the mitochondrial membrane fission/fusion process and mitophagy (28, 31, 51, 52), we assessed if dopaminergic REST deletion modulates Mn-induced dysregulation of fission/fusion and mitophagy processes in the midbrain of mice. Results showed that p62, a mito/autophagy cargo adaptor protein that assists in sequestering damaged organelles and proteins for degradation (53) and indicative of autophagy impairment, were increased in the midbrain of REST-cKO mice compared to the WT mice (Fig. 5*A*). Mn also increased p62 levels in WT mice, with a further increase in REST-cKO mice. In addition, Mn decreased levels of LAMP1, a lysosomal marker, in the midbrain of WT mice, which were further reduced in REST-cKO mice (Fig. 5*A*). LAMP1 was also decreased in REST-cKO mice compared to WT mice. Moreover, Mn increased mitochondrial membrane fission protein Drp1 levels, while it decreased fusion proteins Opa1 and Mfn2 in the midbrain of WT mice, which were more pronounced in REST-cKO mice (Fig. 5*A*). Opa1 and Mfn2 protein levels were also decreased in REST-cKO mice compared to the WT mice. These proteins were assessed in the midbrain tissue homogenates. This is based on the previous reports that although mitochondrial fractions are generally used to detect these fission/fusion proteins (54), the whole tissues showed similar trends (55). Corroborating with these results, IHC imaging showed that Mn also increased fluorescent signals of Drp1 and decreased Mfn2 in mitochondria, with TOM20, a marker of mitochondrial mass and metabolic function (56), in the substantia nigra of WT mice, with further exacerbation in REST-cKO mice (Fig. 5*B*).

Analogous results were also seen in mitophagy-related proteins, parkin and PINK1, as Mn increased their levels in the midbrain of WT mice, which were more pronounced in REST-cKO mice (Fig. 5*A*). The protein levels of PINK1 and parkin were measured in the midbrain tissue homogenates. Although these proteins are commonly measured in mitochondrial fractions as they are recruited to mitochondria upon the activation signals (57) for the mitophagy assessment, whole tissues showed similar results (52, 58, 59) and were used for the present study.

We also examined if mRNA levels of these mito/autophagy proteins were altered by Mn or dopaminergic REST deletion. Mn increased p62 mRNA levels but decreased LAMP1 mRNA levels in the midbrain of WT mice, which were more pronounced in REST-cKO mice (Fig. 5*C*), exhibiting similar trends to those observed in the protein levels, indicating the possible transcriptional modulation of these genes. Mn increased parkin mRNA levels in WT mice, but dopaminergic REST deletion did not exacerbate this effect, unlike protein levels. Mn did not alter PINK1 mRNA levels in both WT and cKO mice, but REST-cKO mice increased basal PINK1 mRNA levels compared to WT mice, showing independent effects of Mn (Fig. 5*C*).

**Figure 6:**
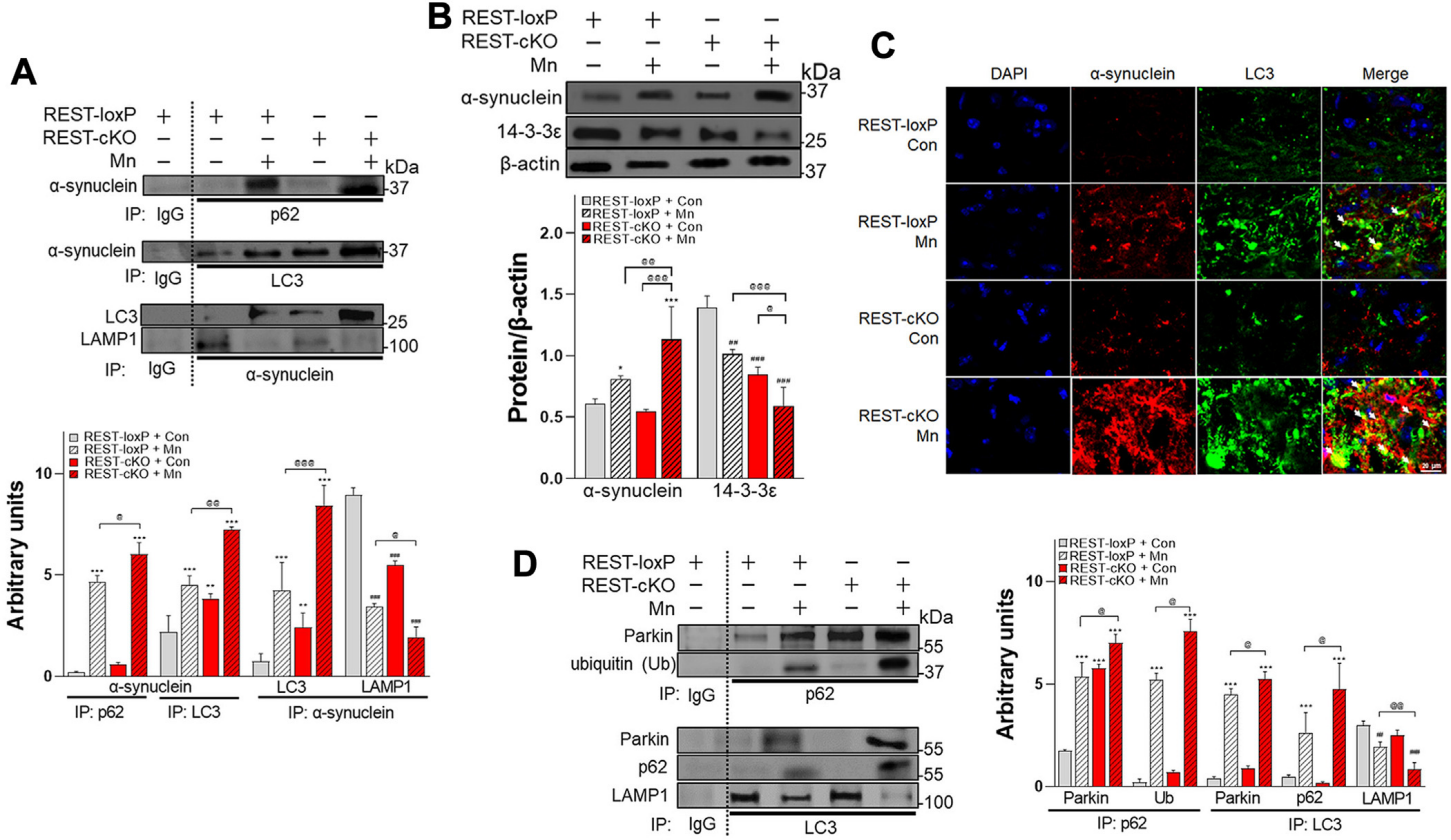
**Deletion of dopaminergic REST deletion exacerbates Mn-induced increased accumulation of α-synuclein and disruption of mitophagy in the midbrain of mice.***A*, protein interactions between α-synuclein, p62, LC3, and LAMP1 were conducted by co-IP in the midbrain tissues of mice. *B*, protein levels of α-synuclein and 14-3-3ε were assessed by Western blotting in the midbrain tissues of mice. *C*, coronal sections of the mouse substantia nigra region were immunostained for α-synuclein (*red*) and LC3 (*green*, autophagosome marker). *White* arrows indicate colocalization of α-synuclein and LC3 (autophagosomes). *D*, protein interactions between p62, parkin, and ubiquitin and between p62 or LC3 were conducted by co-IP in the midbrain tissues. β-actin was used as a loading control. ^∗^*p* < 0.05, ^∗∗^*p* < 0.01, ^∗∗∗^*p* < 0.001, ^#^*p* < 0.05, ^##^*p* < 0.01, ^###^*p* < 0.001, compared with the controls; ^@^*p* < 0.05, ^@@^*p* < 0.01, ^@@@^*p* < 0.001, compared with each other (two-way ANOVA with two independent variables of genotypes and Mn treatment, followed by Tukey’s *post hoc* test; n = 3). Data are expressed as mean ± SD.

To better understand the role of dopaminergic REST in Mn-induced impairment of the interactions of mito/autophagy-related proteins and protein accumulation, we conducted co-immunoprecipitation experiments for several relevant proteins including p62 and LAMP1. Notably, Mn increased the interaction of α-synuclein with autophagosome proteins such as LC3 and p62 but decreased its interaction with LAMP1, leading to α-synuclein accumulation (Fig. 6, *A* and *B*), a critical feature of PD pathology. Mn also increased α-synuclein accumulation, colocalizing with LC3-labeled autophagosomes in the mouse substantia nigra with its further accumulation in REST-cKO mice (Fig. 6*C*). In addition, Mn decreased protein levels of 14-3-3ε, an autophagy regulator (60) in the midbrain of WT mice, an effect that was more pronounced in REST-cKO mice (Fig. 6*B*) (60).

Further analysis of the Mn-induced dysregulation of mitophagy revealed that Mn increased interactions of p62 with parkin and ubiquitin in WT mice, showing more pronounced effects in REST-cKO mice (Fig. 6*D*). In addition, interaction of parkin and p62 with the autophagosome marker LC3 were increased in WT mice exposed to Mn, which was exacerbated in REST-cKO mice (Fig. 6*D*). Mn also decreased the interaction of LC3 and LAMP1, indicating impairment in fusion between autophagosome (LC3) and lysosome (LAMP1) in WT mice, an effect which was further reduced in REST-cKO mice (Fig. 6*D*).

### Dopaminergic REST restoration in the substantia nigra attenuates Mn-induced neurotoxicity in REST-cKO mice

**Figure 7:**
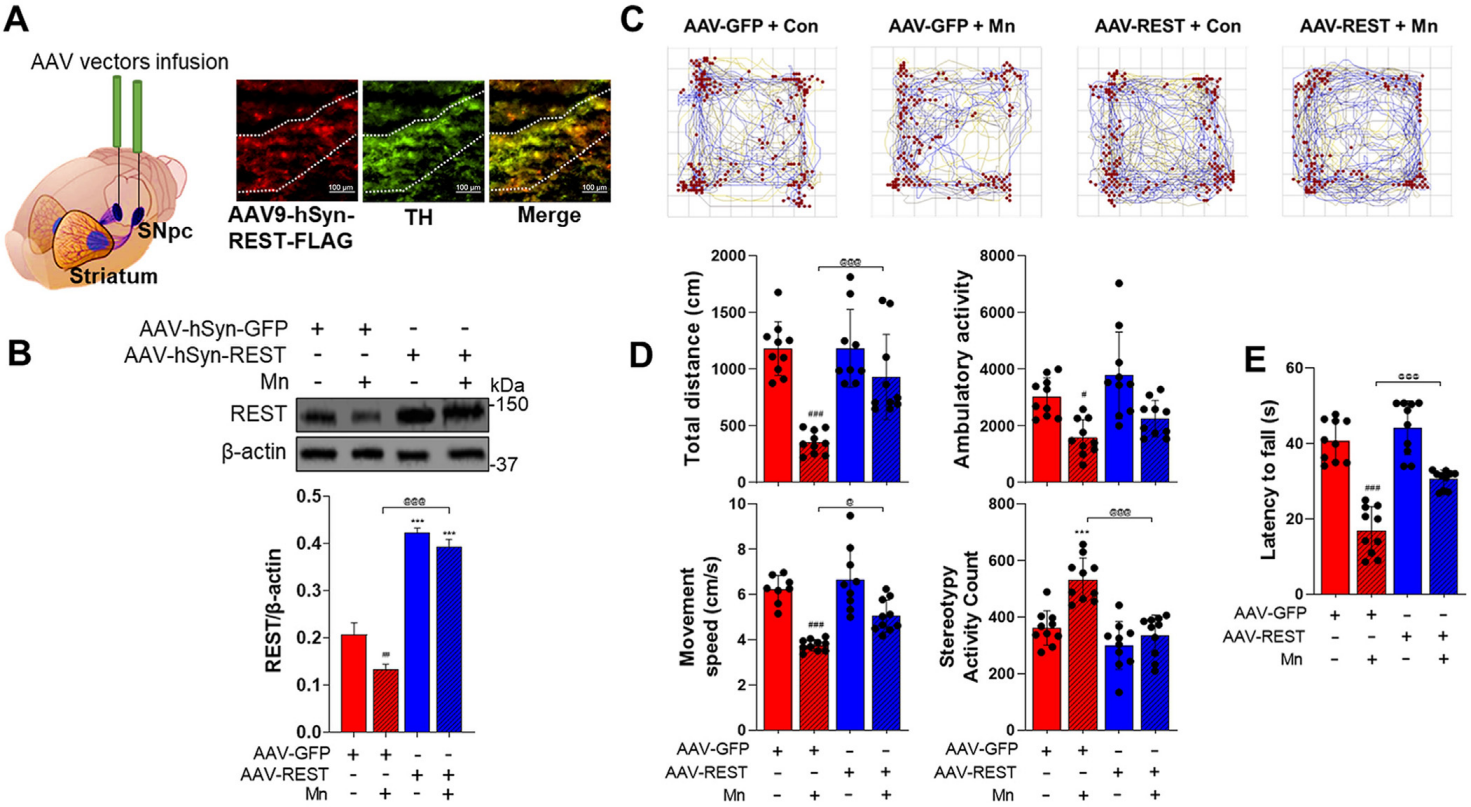
**Neuronal REST restoration in the substantia nigra of REST-cKO mice attenuates Mn-induced deficits in locomotor activity and motor coordination.***A* and *B*, confirmation of REST expression in the dopaminergic neurons in the substantia nigra. Following the viral particle infusion of AAV9-GFP and AAV9-hSYN-REST-FLAG into the substantia nigra, an additional 3 weeks were allowed for the protein expressions in REST-cKO mice. REST (*red*) and TH (*green*) in the substantia nigra of REST-cKO mice were performed by IHC with coronal sections (30 μm) of the REST-cKO mouse brains (*A*). Mn was then treated for 3 weeks as described in the Experimental procedures, followed by an assessment of REST expressions in the midbrain tissues by Western blotting (*B*). *C*, effects of Mn on movements in REST-cKO mice infused with AAV9-GFP and AAV9-REST viral particles were assessed by the traces that depicted the movement of a single mouse with *red* dots denoting vertical activity. *D*, Mn effects on locomotor activities in REST-cKO mice infused with AAV9-GFP and AAV9-REST viral particles were assessed for total distance traveled, horizontal activity, ambulatory/walking activity, movement speed, and stereotypy activity count. *E*, Mn effects on motor coordination in REST-cKO mice infused with AAV9-GFP and AAV9-REST viral particles were assessed by measuring time spent on the rotating rod. ^∗∗∗^*p* < 0.001, ^##^*p* < 0.01, ^###^*p* < 0.001, compared with the controls; ^@^*p* < 0.05, ^@@@^*p* < 0.001, compared with each other (two-way ANOVA with two independent variables of genotypes and Mn treatment, followed by Tukey’s *post hoc* test; n = 10). Data are expressed as mean ± SD.

Since dopaminergic REST deletion exacerbated Mn toxicity, we tested if restoring REST expression in dopaminergic neurons of the midbrain could mitigate Mn-induced neurotoxicity. To achieve this, AAV viral particles were infused into the substantia nigra where dopaminergic cell bodies are localized. AAV9-hSyn-GFP vectors were used as a control, and the AAV9-hSyn-REST-FLAG vectors were used to express REST (Fig. 7, *A* and *B*). REST was tagged with FLAG instead of GFP because GFP was too large to be inserted into the AAV9-hSyn-REST vectors. Three weeks after infusion of the viral particles, mice were exposed to the same Mn exposure paradigm. REST restoration attenuated Mn-induced deficits in locomotor activity and motor coordination (Fig. 7, *C*–*E*) as Mn-induced decrease in total distance traveled, walking activity, and speed were improved in REST-restored substantia nigra of REST-cKO (Fig. 7, *C* and *D*). Mn-induced motor coordination impairment was also attenuated by REST restoration in REST-cKO mice (Fig. 7*E*).

**Figure 8:**
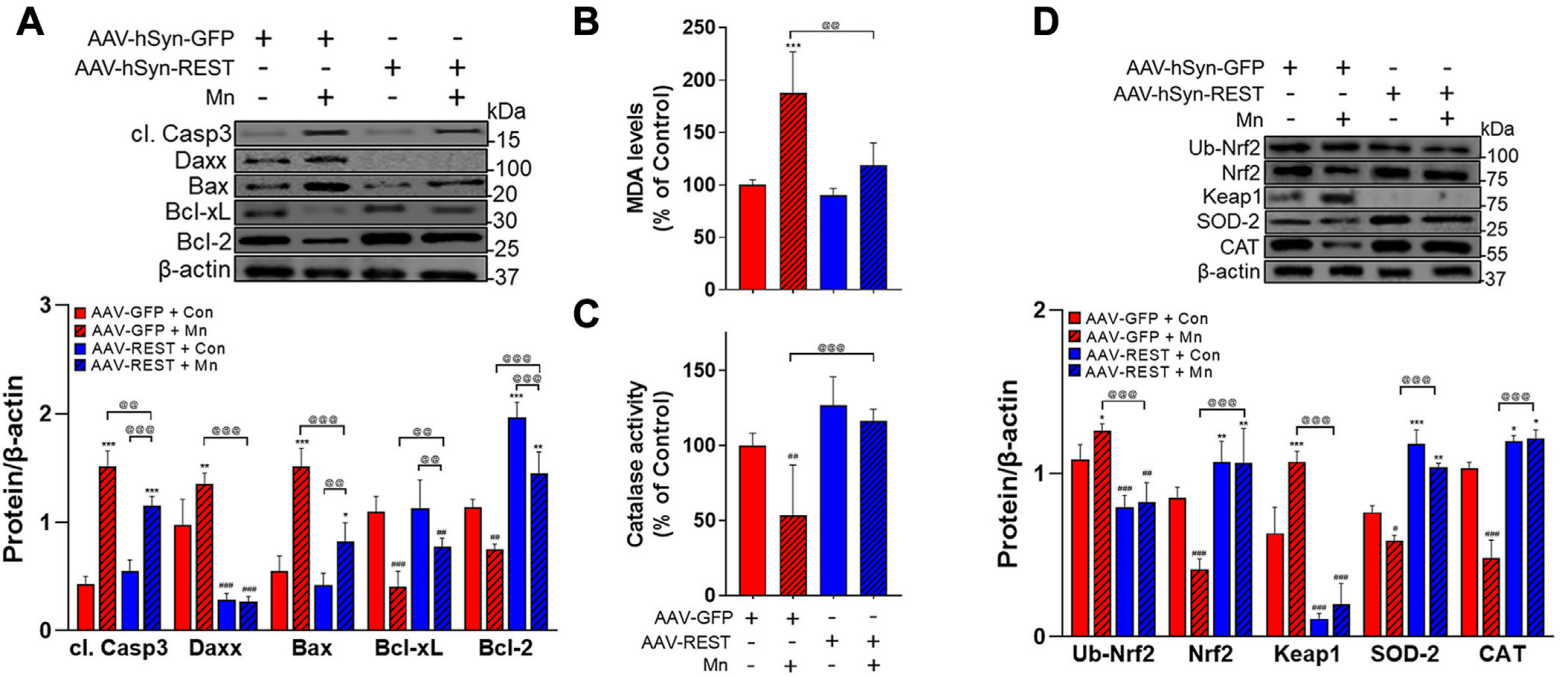
**Neuronal REST overexpression in the substantia nigra of REST-cKO mice attenuates Mn-induced dopaminergic neurotoxicity.***A*, following the viral particle infusion of AAV9-GFP and AAV9-hSYN-REST-FLAG into the substantia nigra, an additional 3 weeks were allowed for the protein expression. Mn was then treated in REST-cKO mice as described in the Experimental procedures, followed by the measurement of protein levels associated with apoptosis in the midbrain. *B* and *C*, MDA levels for lipid peroxidation (*B*) and catalase activity for antioxidative response (*C*) were measured in the midbrain. *D*, oxidative stress-response proteins, such as Nrf2, ubiquitinated Nrf2, Keap1, SOD-2, and catalase (CAT), in the midbrain were assessed by Western blotting. β-actin was used as a loading control. ^∗^*p* < 0.05, ^∗∗^*p* < 0.01, ^∗∗∗^*p* < 0.001, ^#^*p* < 0.05, ^##^*p* < 0.01, ^###^*p* < 0.001, compared with the controls; ^@@^*p* < 0.01, ^@@@^*p* < 0.001, compared with each other (two-way ANOVA with two independent variables of genotypes and Mn treatment, followed by Tukey’s *post hoc* test; n = 3). Data are expressed as mean ± SD.

REST restoration in REST-cKO mice also attenuated Mn-induced dysregulation of apoptotic proteins, such as caspase-3, Daxx, Bax, Bcl-xL, and Bcl-2 levels (Fig. 8*A*). Moreover, REST restoration in the midbrain also mitigated Mn-dysregulated oxidative stress–related proteins as well as indicators including MDA levels (Fig. 8*B*) and catalase activity (Fig. 8*C*). Mn-induced dysregulation on antioxidant-related proteins, such as SOD-2, CAT, Nrf2, ubiquitinated Nrf2, and Keap1, were restored nearly to the control levels by REST restoration in the midbrain of REST-cKO mice (Fig. 8*D*).

**Figure 9:**
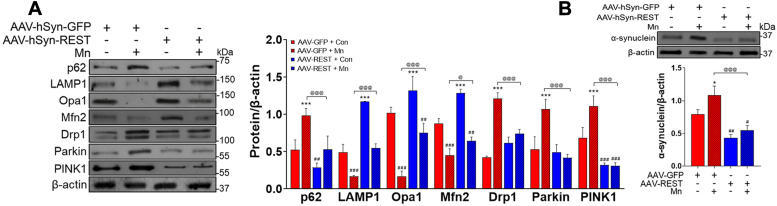
**Neuronal REST overexpression in the substantia nigra of REST-cKO mice attenuates Mn-induced dysregulation of mitochondrial fission/fusion and mitophagy.***A*, following the viral particle infusion of AAV9-GFP and AAV9-hSYN-REST-FLAG into the substantia nigra, allowing for an additional 3 weeks for the protein expression, REST-cKO mice were exposed to Mn as described in the Methods, followed by assessment of protein levels of p62, LAMP1, Opa1, Mfn2, Drp1, parkin, and PINK1 in the midbrain. *B*, protein levels of α-synuclein were measured by western blotting. β-actin was used as a loading control. ^∗^*p* < 0.05, ∗∗∗*p* < 0.001, ^#^*p* < 0.05, ^##^*p* < 0.01, ^###^*p* < 0.001, compared with the controls; ^@^*p* < 0.05, ^@@^*p* < 0.01, ^@@@^*p* < 0.001, compared with each other (two-way ANOVA with two independent variables of genotypes and Mn treatment, followed by Tukey’s *post hoc* test; n = 3). Data are expressed as mean ± SD.

Restoration of the REST protein in the substantia nigra of REST-cKO mice attenuated the Mn-induced dysregulation on the mitochondrial fission/fusion and mitophagy pathway by restoring mitochondrial fission/fusion proteins such as Drp1, Opa1, and Mfn2 and mitophagy proteins such as p62, parkin, PINK1, and LAMP1 (Fig. 9*A*). REST restoration also attenuated Mn-induced α-synuclein accumulation (Fig. 9*B*).

### Role of REST in Mn-induced toxicity associated with mitochondrial damage in neuronal cultures

**Figure 10:**
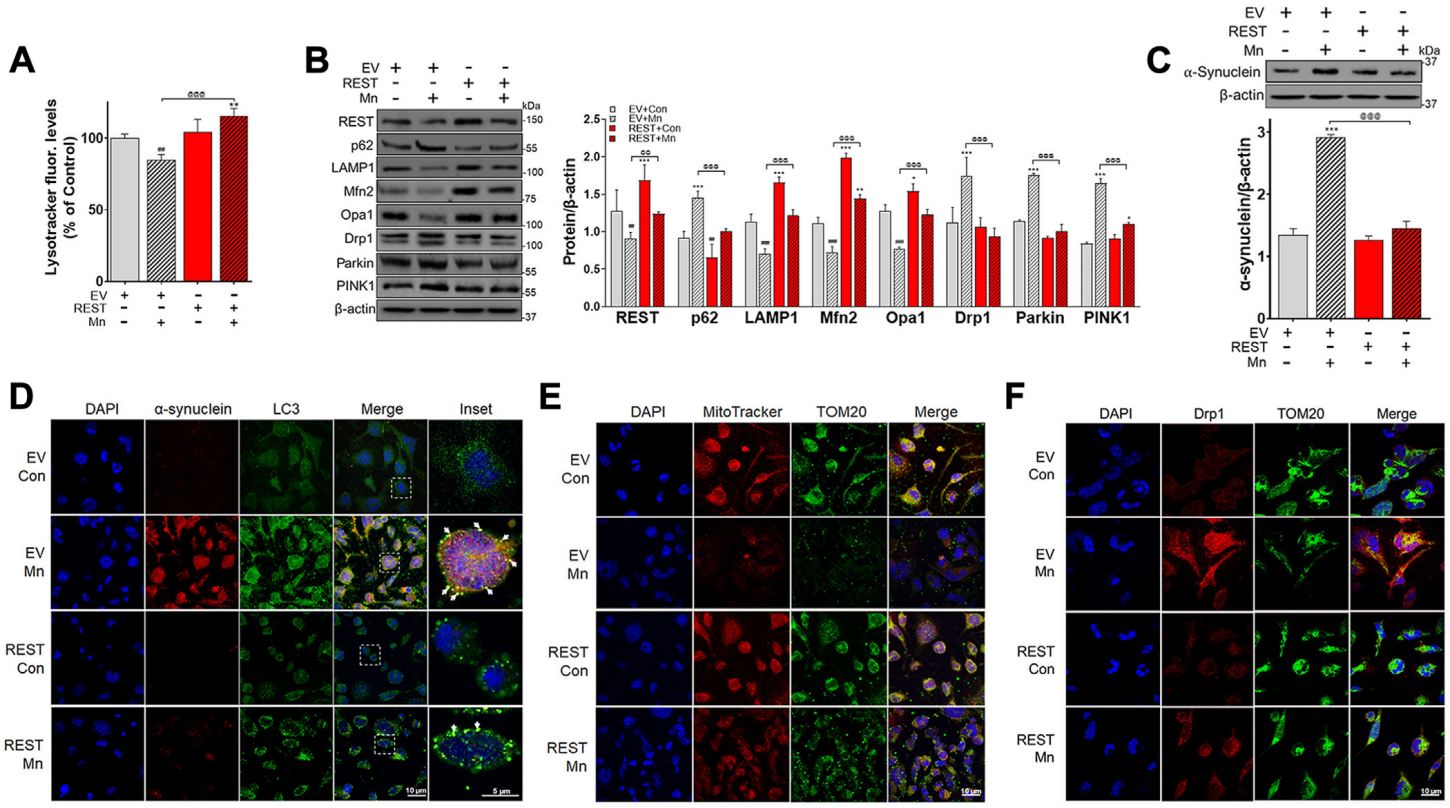
**REST overexpression attenuates Mn-induced dysregulation of mitochondrial fission/fusion and mitophagy in CAD neuronal cells.***A*, cells were transfected with EV and REST, followed by Mn exposure (Mn, 250 μM, 12h), and the lysosomal function was assessed by the lysotracker assay. *B* and *C*, protein levels of REST, p62, LAMP1, Mfn2, Opa1, Drp1, parkin, and PINK1 (*B*), and α-synuclein (*C*) were measured by Western blotting. β-actin was used as a loading control. *D*, CAD cells were immuno-stained for α-synuclein (*red*) and LC3 (*green*, autophagosome marker). *White* arrows indicate the subcellular colocalization of α-synuclein and LC3 (autophagosomes). Insets show a magnified view of the cell and colocalization of α-synuclein and LC3. *E* and *F*, CAD cells were assessed for mitochondrial functions by ICC imaging of MitoTracker or fission protein Drp 1 (*red*) and TOM20 (*green*, a mitochondrial marker). ^∗^*p* < 0.05, ^∗∗∗^*p* < 0.001, ^#^*p* < 0.05, ^##^*p* < 0.01, ^###^*p* < 0.001, compared with the controls; ^@^*p* < 0.05, ^@@@^*p* < 0.001, compared with each other (two-way ANOVA with two independent variables of genotypes and Mn treatment, followed by Tukey’s *post hoc* test; n = 3–6). Data are expressed as mean ± SD. The data are representative of three independent experiments.

Next, we used an *in vitro* neuronal culture model to confirm the role of REST in Mn toxicity by REST overexpression. Lysotracker detection and expression of mitochondrial dynamics and mitophagy proteins were assessed in Cath.a-differentiated (CAD) cells. The results showed that Mn decreased lysotracker fluorescence intensity in empty vector (EV)-transfected CAD cells but was attenuated in REST-overexpressing CAD cells (Fig. 10*A*). In addition, Mn dysregulated the protein expressions of mitochondrial fission/fusion (Mfn2, Opa1, Drp1) and mitophagy (parkin, PINK1, p62, LAMP1) in control CAD cells, while these effects were attenuated in REST-overexpressing CAD cells (Fig. 10*B*). Moreover, Mn increased accumulation of α-synuclein but attenuated by REST overexpression in CAD cells (Fig. 10*C*). In corroborating with these results, immunocytochemistry (ICC) data showed that Mn-induced α-synuclein accumulation colocalized with LC3-labeled autophagosomes in EV-transfected CAD cells, while REST overexpression attenuated these effects (Fig. 10*D*). Moreover, Mn caused mitochondrial damage, showing a decrease in mitochondrial membrane potential with MitoTracker and an increase in the mitochondrial fission protein Drp1 in EV-transfected CAD cells, an effect attenuated by REST overexpression (Fig. 10, *E* and *F*).

## Discussion

**Figure 11:**
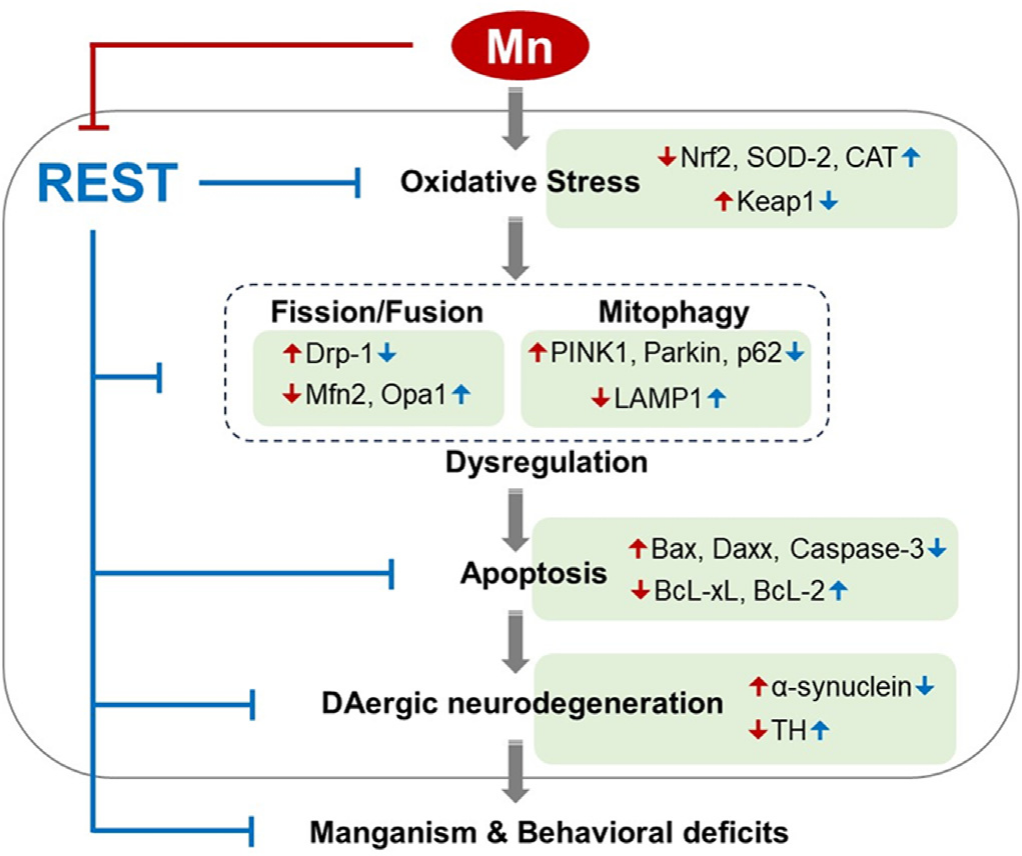
**A proposed mechanism outlining the REST’s role in protecting dopaminergic neurons against Mn-induced neurotoxicity.** The transcription factor REST (*blue* lines) protects dopaminergic neurons by counteracting the Mn-induced increases in pro-apoptotic factors and decreases in anti-apoptotic and anti-oxidative defense mechanisms within dopaminergic neurons. Mn (*red* lines) triggers oxidative stress, autophagy/mitophagy dysfunction, and apoptosis in midbrain dopaminergic neurons. These detrimental effects can be attributed, at least in part, to Mn’s reduction of dopaminergic REST, amplifying its toxic impact within the cell. Mn-induced auto/mitophagy impairment leads to the accumulation of damaged and toxic substances, further contributing to dopaminergic neurotoxicity. This proposed mechanism sheds light on the intricate interplay between REST and Mn in the context of dopaminergic neuroprotection.

The findings from the present study demonstrate that dopaminergic REST plays a critical role in attenuating Mn-induced neurotoxicity, including apoptosis, oxidative stress, and impairment of mitophagy in both mouse brain and neuronal cultures (Fig. 11). These findings are supported by observations that dopaminergic REST deletion exacerbated Mn-induced neurotoxicity, while restoration of REST with AAV-REST viral vectors in REST-cKO mice attenuated Mn toxicity, along with REST-associated rescue from Mn neurotoxicity in neuronal cultures. These findings suggest that dopaminergic REST could be a putative target to mitigate Mn-induced neurotoxicity, providing potential therapeutic strategies.

We have generated, for the first time, a dopaminergic-specific REST-cKO mouse model by crossing REST loxP mice with DAT-Cre mice, which expresses Cre recombinase in dopaminergic neurons (DAT-expressing cells), resulting in the deletion of REST specifically in dopaminergic neurons. To understand the role of dopaminergic REST in Mn toxicity, this mouse model offers significant advantages over previous neuron-specific REST-cKO models, which used nestin- or neuron-specific enolase for pan-neuronal REST deletion (39, 40, 61). The REST-cKO mice were healthy and showed no abnormalities in their development and growth prior to starting the experiments, suggesting that this REST-cKO mouse model is highly relevant to study the role of dopaminergic REST in Mn-induced neurotoxicity. Since Cre recombinase is expressed under the DAT promoter control, in addition to substantia nigra, other dopaminergic neurons, such as those localized in the ventral tegmental area in the midbrain, will also express Cre recombinase (62, 63). However, Mn is known to accumulate preferentially in the basal ganglia, resulting in motor deficits mainly associated with the nigrostriatal pathway. Therefore, we have focused on the nigrostriatal dopaminergic pathway in the present study. The role of REST in other dopaminergic pathways remains to be explored. Our experimental design for Mn exposure in mice increased Mn levels in the mouse brain by about 2-fold, which is highly relevant to Mn exposure levels in humans as it increases up to 3-fold (64), and impaired dopaminergic function and neuronal toxicity (40, 65). Our findings that dopaminergic REST deletion further exacerbated Mn toxicity in mice and neuronal cultures highlight the role of dopaminergic REST in protecting against Mn toxicity.

We have previously reported that Mn decreased REST expression, while REST enhanced the expression of TH in dopaminergic LUHMES and CAD cell models (18), mitigating the Mn-induced reduction in TH protein levels. The present study in an *in vivo* animal model provides additional evidence for the role of dopaminergic REST in protecting against Mn toxicity. Since REST exerts protective effects in neurodegenerative disorders such as AD and PD (38, 40), it may have broad protective mechanisms and be a highly relevant molecular target for developing therapeutics for neurodegenerative diseases. While REST plays a role as a neuroprotectant and is decreased in many neuropathological conditions (66), one study showed that REST levels were increased in a PD model induced by the neurotoxin MPP^+^ in SH-SY5Y cells (67), possibly due to a compensatory mechanism. Thus, additional research is warranted to better understand time- and context-dependent mechanisms of REST modulation by neurotoxins.

Dopaminergic REST-cKO mice showed no significant behavioral deficits, except for stereotypy activity (Fig. 2). REST-cKO mice did not affect the developmental stage nor normal growth in mice, but global REST deletion led to embryonic lethality (68), indicating that REST is essential for development and survival. In line with the role of REST on survival, increased REST expression is correlated with longevity and protective roles in aging (40, 65). Interestingly, dopaminergic REST deletion altered stereotypy activity (Fig. 2*C*), exhibiting repetitive movements. Dysregulation of REST is often observed in various neurological disorders (69), and increased stereotypical behaviors were observed in AD, frontotemporal dementia, PD with dementia, and autism in clinical and animal models (69, 70, 71), requiring further studies to understand a potential link between impairment in REST and stereotypical behaviors in these neurological disorders. Moreover, the Mn-induced behavioral deficits, including impairment in locomotor activity and motor coordination, were more pronounced in dopaminergic REST-cKO mice (Fig. 2), demonstrating that dopaminergic REST is involved in Mn-induced motor dysfunction. This is further supported by the results showing that REST restoration in the substantia nigra of REST-cKO mice significantly attenuated these Mn-induced behavioral deficits (Fig. 7).

Several potential mechanisms may underlie REST’s neuroprotective effects against Mn toxicity. REST as a TF regulates the expression of genes involved in oxidative stress responses and apoptosis (40, 72), as well as mitochondrial function (73, 74), which are directly associated with mechanisms that mediate Mn toxicity. This suggests that REST’s protection and Mn’s toxicity share common molecular targets, providing a critical role of REST in preventing Mn toxicity. Moreover, REST’s mitigation of Mn-induced dysregulation of mitophagy-associated protein expression, including p62, LAMP1, parkin, and PINK1 (Fig. 9*A*), suggests that REST might be critical for protecting against Mn-induced dysregulation of mito/autophagy function. Future studies are required to better understand the precise molecular mechanisms underlying REST’s protection against Mn toxicity.

REST plays a critical role in mitigating Mn-induced apoptosis in dopaminergic neuronal cell cultures (18), and the present *in vivo* study showed that Mn-induced apoptosis in WT mice was further exacerbated in REST-cKO mice (Fig. 3). These findings in dopaminergic REST-cKO are supported by previous studies that showed increased vulnerability of mice with neuron-specific REST deletion to MPTP (a PD neurotoxin) toxicity (39, 61), suggesting a broader protective role of neuronal REST against multiple neurotoxicants. Moreover, single-cell RNA-seq analysis from the brains of PD patients reveals a link between REST dysregulation and apoptosis in dopaminergic neurons (75). Specifically, REST as a TF may directly target pro- or anti-apoptotic genes, such as Daxx and Bcl-2, by binding to their promoters (76).

The present study provides compelling evidence that REST enhances antioxidant defense mechanisms in Mn-induced neurotoxicity as REST restoration mitigated the Mn-induced reduction of antioxidant proteins and increase of oxidative activities in the midbrain of REST-cKO mice (Fig. 8). These *in vivo* results corroborate our previous findings from *in vitro* LUHMES and CAD neuronal cell cultures (18), underscoring the role of REST in antioxidant defenses. In fact, REST is known to regulate the expression of a wide array of genes involved in antioxidant responses (77), including catalase and SOD-2 levels, by direct interaction with their target gene promoters (18). Moreover, REST also modulates the Nrf2 antioxidant response system through various transcription factors or the ubiquitin-proteasome system, notably *via* Keap1 (18, 78). Mn effects on oxidative stress responses in Nrf2-Keap1 signaling by decreasing Nrf2 and increasing Keap1 protein levels were corroborated with the previous study showing the decreased nuclear Nrf2 and increased Keap1 protein levels in rotenone-induced PD model in mice (79). Nonetheless, the precise mechanisms by which REST regulates the Nrf2 pathway require further investigation.

Mn-induced impairment in autophagy and mitophagy (10, 17, 26, 27, 28) is closely associated with dopaminergic REST and Mn-induced dopaminergic neurotoxicity. Mitochondrial fission/fusion and mitophagy processes are closely working with the autophagy process by sharing many autophagy process-associated proteins, including Beclin 1 (34, 80), to remove defective mitochondria from the cell (80). Mn-induced dysregulation of mito/autophagy may cause the accumulation of α-synuclein oligomers and neuronal damage in mice (26). Studies have shown that enhancing autophagy with trehalose, resveratrol, or curcumin mitigated Mn toxicity in rats, mice, and PC12 cells (26, 27, 28). However, the role of REST in Mn toxicity, particularly *via* mito/autophagy regulation, has not been studied. Our findings demonstrate that dopaminergic REST plays a critical role in mitophagy process as its deletion exacerbated Mn-induced dysregulation of mitophagy-associated proteins (Fig. 5) and further accumulated α-synuclein in the midbrain of mice (Fig. 6*B*). It has been reported that REST is essential for normal autophagy function (38, 81) and prevents the senescence process as REST deficiency impairs mito/autophagy and leads to cellular senescence in primary mouse neurons (81).

Expression levels of proteins involved in initiating mitophagy, such as PINK1 and parkin, were increased in REST-cKO and Mn exposure (Fig. 5), accompanied by higher interaction between parkin and either p62 or LC3 (Fig. 6*C*), suggesting the activation of initial stages of mitophagy flux and accumulation of damaged proteins in autophagosomes. However, the mito/autophagy process is disrupted at the autophagy-lysosomal fusion step, as evidenced by reduced interaction between LC3 and LAMP1 (Fig. 6*C*), consistent with impaired lysosomal degradation capacity and ultimate autophagy impairment. This was further supported by p62 accumulation (Fig. 5*A*), which indicates impaired autophagy, in addition to its role as a key cargo adaptor in the autophagy process (82). Moreover, Mn dysregulated the mitochondrial membrane fission/fusion process by upregulating fission protein Drp1 and downregulating fusion proteins such as Opa1 and Mfn2, which were further pronounced in REST-cKO mice (Fig. 5*A*), potentially contributing to mitophagy initiation (83). However, later steps of mitophagy, which involve the fusion of autophagosomes and lysosomes, were compromised by Mn or REST deletion, resulting in impaired function of mitophagy/autophagy and accumulation of proteins such as α-synuclein. The accumulation of α-synuclein in autophagosomes is closely associated with aberrant protein degradation in neurodegenerative diseases (61, 84, 85).

Restoring REST expression in the substantia nigra by infusions of AAV9-hSYN-m-REST viral particles mitigated Mn-induced dopaminergic neurotoxicity, including behavioral deficits (Fig. 7), establishing that dopaminergic REST is critical in protecting against Mn toxicity. Since dopaminergic cell bodies are localized in the substantia nigra and REST is likely incorporated into the nucleus of dopaminergic neurons, REST may regulate the expression of target genes related to apoptosis and oxidative stress by either activating or repressing target genes (43). These suggestions are supported by previous findings that REST modulated several transcriptional networks involved in neuroprotection and neurotoxicity, such as FOXO1 and YY1 (44, 65, 81, 86). FOXO1 was activated by REST and promoted antioxidant defense mechanisms, longevity, and autophagy in mouse primary neurons (18, 65, 81). On the other hand, YY1, which was upregulated by Mn and decreased GLT-1 in astrocytes (87, 88), repressed REST, thereby promoting Mn toxicity (44). These findings suggest that REST affords protective effects regardless of neural cell types in the brain. While the present study demonstrates that dopaminergic REST in the nigrostriatal regions plays a critical role in protecting against Mn-induced neurotoxicity, a potential limitation of the REST-cKO mouse model is that REST deletion may also occur in other brain regions that contain DAT-expressing cells, particularly VTA regions of the midbrain, where dopaminergic cell bodies are also localized to innervate the limbic and mesocortical pathways (89). Nonetheless, VTA-originating dopaminergic REST did not affect REST’s protective effect against Mn toxicity, likely because Mn primarily targets the basal ganglia (90). In addition, REST expression in dopaminergic neurons and astrocytes attenuates Mn toxicity (11, 44), warranting further investigation to better understand REST’s protective mechanisms in different neural cell types.

In summary, dopaminergic REST plays a pivotal role in protecting against Mn-induced neurotoxicity by reducing oxidative stress, mitigating apoptosis, enhancing antioxidative mechanisms, and restoring the mitophagy processes and their related genes (Fig. 11). Our findings underscore dopaminergic REST's essential role in maintaining a regulatory network for neuroprotection against Mn toxicity and potentially other neurodegenerative diseases.

## Experimental procedures

### Chemicals, antibodies, and reagents

MnCl_2_, dimethyl sulfoxide, resazurin sodium salt (R7017), and REST antibody (07-579) were purchased from MilliporeSigma. Antibodies for REST (sc-374611), TH (sc-25269), caspase-3,(sc-7272), Bax (sc-7480), Daxx (sc-8043), Bcl-xL (sc-8392), Bcl-2 (sc-7382), SOD-2 (sc-137254), catalase (CAT, sc-271803), Ub-Nrf2/Nrf2 (sc-722), Keap1 (sc-365626), p62/SQSTM1 (sc-28359), LAMP1 (sc-20011), Opa1 (sc-393296), Mfn2 (sc-515647), Drp1 (sc-271583), PINK1 (sc-518052), parkin (sc-32282), ubiquitin (sc-8017), α-synuclein (sc-12767), 14-3-3ε (sc-23957), TOM20 (sc-17764), and β-actin (sc-47778) were obtained from Santa Cruz Biotechnology. Antibodies for Cre recombinase (ab216262), LC3B (ab48394), rabbit anti-mouse (ab6728), goat anti-rabbit (ab97051) conjugated with horseradish peroxidase, and goat anti-rabbit and anti-mouse antibodies conjugated with Alexa Fluor 488, 568, or 647 were obtained from Abcam. Antibody for TOM20 was purchased from Cell Signaling Technology. AAV particles for AAV9-hSYN-m-REST-FLAG and AAV9-hSYN-m-GFP vectors were purchased from Vector Biolabs. Plasmid DNA for REST-myc and pCMV6-entry empty vectors were purchased from OriGene. Catalase activity colorimetric/fluorometric kit (Cat. #K773-100) was obtained from BioVision. MDA/lipid peroxidation/TBARS assay kit (Cat. # 10009055) was obtained from Cayman Chemical. MitoTracker Red (M7512) and Lysotracker Deep Red (L12492) were purchased from Invitrogen.

### Mn treatment and AAV infusion in mice

Dopaminergic neuron-specific REST KO mice were generated by crossing REST-loxP mice with DAT-Cre (DAT^IRES.*Cre*^) mice in our institutional animal facility, as shown in Figure 1*A* (47). DAT-Cre mice were purchased from Jackson Lab (Strain #: 006660), and REST loxP mice (Jackson Lab, strain #:024549) were obtained from Dr Jenny Hsieh, Southwestern Medical Center. All animal research protocols were approved by the Institutional Animal Care and Use Committee at Florida A&M University (FAMU). Mice were kept in groups of five per cage, maintaining a 12-h light/dark cycle at a constant temperature of 22 ± 2 °C with ad libitum access to food, water, and enrichment. A total of 80 male mice were used in the first experiment. Twenty mice were randomly divided into four groups and were designated as follows: (1) REST-loxP/WT plus vehicle, (2) REST-loxP/WT plus Mn, (3) REST-cKO plus vehicle, and (4) REST-cKO plus Mn. The mice were treated daily with Mn at a dose of MnCl_2_ 30 mg/kg (330 μg of Mn, 1 μl per nostril in both nostrils) for 3 weeks. This dosage increases brain Mn levels by up to twofold in mice (91), which is relevant to pathophysiological Mn levels of about threefold increase observed in nonhuman primate brains affected by Mn toxicity (64, 92, 93). Distilled water was used as a vehicle. To prevent Mn expulsion from the nostrils, the mice were sedated with isoflurane for 3 min before and after Mn instillation.

For REST overexpression experiments, 40 REST-cKO male mice were used for AAV infusion. AAV particles for the control AAV9-hSYN-m-GFP and AAV9-hSYN-m-REST-FLAG vectors were purchased from Vector Biolabs. These mice were randomly divided into four groups with the following designations: (1) AAV9-hSYN-m-GFP plus vehicle, (2) AAV9- hSYN-m-GFP plus Mn, (3) AAV9-hSYN1-m-REST-FLAG plus vehicle, and (4) AAV9-hSYN-m-REST-FLAG plus Mn. To ensure precise AAV infusion, mice were anesthetized using ketamine/xylazine (100 mg/kg) and secured in a stereotaxic apparatus. One microliter of viral vectors was infused into both sides of the substantia nigra of the mouse brain using a 10-μl Hamilton syringe (Stoelting Inc.) connected to a microinjection pump. The infusion was administered at a steady flow rate of 0.2 μl/min. The stereotaxic injection coordinates for the substantia nigra were established as follows: (measured from bregma) x = ±1.2 mm (medial-lateral), y = −3.0 mm (anterior-posterior), and z = −4.3 mm (dorsal-ventral). The needle was left in place for an additional 5 min before being slowly withdrawn to prevent the reflux of viral vectors. Following this, surgical incisions were meticulously closed with sutures. Subsequently, mice were kept warm with a heating pad until recovered and then transferred to their respective cages for another 3 weeks to allow for adequate expression of REST protein in the substantia nigra. All mice underwent the same paradigm for Mn exposure, followed by behavioral tests, perfusion, and brain tissue dissection for subsequent biochemical analyses.

### Locomotor activity and motor coordination

Behavioral tests were conducted 24 h after the last Mn exposure to evaluate the effects of Mn as previously described (91). The open-field and rotarod tests assessed locomotor activity and motor coordination, respectively. The open-field test was conducted in a Plexiglas arena, and the movement of each mouse was tracked using Fusion SuperFlex software v6.25. Prior to testing, mice were acclimated to the arena for three consecutive days. The distance traveled, speed, and vertical activity for each mouse were recorded for 30 min, and these parameters were compared between groups using statistical analysis.

The AccuRotor rotarod system was used to assess motor coordination for the rotarod test. Mice were trained for three consecutive days with three trials per session, and the latency to fall was recorded using Fusion Software v6.3. The measurements of motor coordination were taken for 650 s in mice that remained on the rod throughout the test, and the average duration for each group was used for comparisons.

### Cell culture and transfection

All cells were maintained at 37 °C in a 95% air and 5% CO_2_ incubator. For the experiments on neuronal cells, mouse CAD cells (#08100805, MilliporeSigma) were used. Cell culture, maintenance, and transfection were performed according to the established protocol (18). Briefly, CAD cells were cultured using complete growth media (DMEM/F-12 supplemented with 10% fetal bovine serum, 1× GlutaMAX, 100 units/ml penicillin, and 100 μg/ml streptomycin). Cells were incubated with serum-free media for at least 3 days to induce differentiation. Afterward, cells were transfected with the relevant plasmid DNA vectors, followed by Mn treatment (250 μM, 12 h).

Cells were cultured for a density of 2 × 10^7^ per transfection for transfections. Twenty micrograms of plasmid DNA for empty vectors and REST were used for transfection in a 4-mm electroporation cuvette. The electroporation settings were set to 180 V, 950 μF capacitance, and infinite (∞) ohm resistance. Electroporated cells were immediately resuspended in complete growth media and allowed to incubate overnight. Subsequently, the complete growth media was replaced to facilitate further growth over a 48 to 72 h period. Transfections were carried out using the GenePulser Xcell electroporation system (Bio-Rad), following the manufacturer’s instructions.

### Western blotting

Protein samples were collected from brain tissues and cell extracts for protein analysis by immunoblotting. After homogenization in a radioimmunoprecipitation assay buffer with protease inhibitors, the protein concentration was determined by a bicinchoninic acid assay. Equal amounts of protein were loaded onto 10% SDS-PAGE and analyzed by immunoblotting using antibodies at a dilution ranging from 1:500 to 1:1000, followed by horseradish peroxidase–conjugated secondary antibody at a dilution of 1:5000. The blots were developed using the West Pico PLUS chemiluminescence substrate detection kit and imaged using the Bio-Rad ChemiDoc Imaging System. The targeted protein bands were quantified using the Image Laboratory Software version 5.2.1 (Bio-Rad).

### Co-immunoprecipitation

Co-immunoprecipitation experiments were performed based on our previous studies with minor modifications (18, 44, 91). Briefly, 300 μg of protein extracts from the midbrain were mixed with 2.5 μg of specific antibodies, such as p62, LC3, α-synuclein, parkin, ubiquitin, and LAMP1. The mixture was then gently agitated at 4 °C for 2 h. Next, 20 μl of protein A + G-agarose beads (from Santa Cruz) were added, and the mixture was incubated overnight at 4 °C with rotating. Next, the beads were washed thrice with PBS buffer and then eluted with radioimmunoprecipitation assay buffer. Subsequently, 40 μl of sample loading buffer was introduced, and the solution was heated to 95 °C for 10 min. After centrifugation, the resulting eluted proteins in the supernatant were employed for SDS-PAGE and Western blotting.

### ICC and IHC

For ICC, CAD cells were plated on poly-l-lysine–coated glass coverslips in 6-well plates. After treatment of designated compounds, cells were fixed with 4% paraformaldehyde in PBS and followed the established protocol (17) to assess mitochondrial damage with MitoTracker and detection of α-synuclein and LC3 (1:100 dilution) in mitochondria with TOM20 as a mitochondrial marker. After incubation of fluorescent-conjugated secondary antibodies Alexa Fluor 488 and 568 (1:1000 dilution), cellular localization and fluorescence intensity of proteins were detected using a Leica SPEII confocal microscope (Leica Microsystems Inc.).

IHC procedures were performed with minor modifications as previously described (91). Fixed and snap-frozen mouse brain tissue samples were processed to obtain coronal sections of substantia nigra (−2.7 to −3.4 mm) from the bregma, sliced into 30 μm thickness. IHC was conducted to assess the protein expressions of REST, TH, caspase 3, Drp1, Mfn2, TOM20, α-synuclein, and/or LC3 in the substantia nigra, with tissue sections from three mice per group. Antibodies of REST, TH, caspase 3, Drp1, Mfn2, TOM20, α-synuclein, and/or LC3 were diluted 1:250 in the blocking buffer. The IHC on the coronal sections of the substantia nigra of the brain tissues followed the standard protocol (17) and secondary antibodies conjugated with Alexa Fluor 488, 568, and/or 647 fluorescent dyes diluted (1:1000). Protein expressions from the same histological site of each sample were evaluated for fluorescence intensity using a Ts2R fluorescence microscope (Nikon Instruments) and a Leica SPEII confocal microscope (Leica).

### Assays for lipid peroxidation, catalase activity, and lysosomal activity

The assessment of lipid peroxidation and catalase activity followed the manufacturer’s instructions. Endpoint fluorescence in these assays was quantified using the Spectramax i3x multi-mode microplate reader from Molecular Devices. Briefly, midbrain tissues were lysed with a mammalian cell lysis buffer, then the extracts were further analyzed with the specific assay kits. For lipid peroxidation, a TBARS assay kit was used. In brief, tissue lysates were measured for MDA, a by-product of lipid peroxidation that forms a complex upon reacting with thiobarbituric acid. The fluorescence was measured at an excitation/emission wavelength of 530/550 nm using an MDA standard curve for quantification. For catalase activity, tissue lysates were measured for the rate of breakdown in hydrogen peroxide (H_2_O_2_) by measuring the amount of unreacted H_2_O_2_ (using OxiRed Probe) at an excitation/emission wavelength of 535/587 nm. Lysosomal function in CAD cells was measured with Lysotracker Deep Red for 30 min and subsequently measured the fluorescence in the near-infrared spectrum at wavelengths of 647/668 nm.

### Reverse transcription-quantitative PCR

Total RNA was extracted from three samples per group using the RNeasy Mini Kit (Qiagen). Purified RNA (2 μg) was reverse-transcribed into cDNA using the high-capacity cDNA reverse transcription kit (Applied Biosystems). Real-time qPCR was performed on the CFX96 (Bio-Rad) using iQ SYBR Green Supermix (Bio-Rad) and 0.4 μM primers. The total reaction volume was 25 μl, with each cDNA template in 1 μl. The following primers were used for RNA extracted from mouse brain tissues: mouse p62, 5′-AGG ATG GGG ACT TGG TTG C-3′ (forward) and 5′-TCA CAG ATC ACA TTG GGG TGC-3′ (reverse); mouse LAMP1, 5′-CAG CAC TCT TTG AGG TGA AAA AC-3′ (forward) and 5′-ACG ATC TGA GAA CCA TTC GCA-3′ (reverse); mouse parkin, 5′-TCT TCC AGT GTA ACC ACC GTC-3′ (forward) and 5′-GGC AGG GAG TAG CCA AGT T-3′ (reverse); mouse PINK1, 5′-GAG CAG ACT CCC AGT TCT CG-3′ (forward) and 5′-GTC CCA CTC CAC AAG GAT GT-3′ (reverse); mouse GAPDH, 5′-CTC ATG ACC ACA GTC CAT GC-3′ (forward) and 5′-CAC ATT GGG GGT AGG AAC AC-3’ (reverse). GAPDH was used as an internal control. The qPCR parameters were set for 1 cycle at 95 °C for 10 min, 40 cycles at 95 °C for 15 s, and 60 to 65 °C for 1 min. Expression levels of each target gene were detected and quantified using the Bio-Rad CFX Manager version 3.1.

### Statistical analysis

The results are expressed as the mean ± SD. Data analysis was conducted using GraphPad software version 9 (GraphPad). Bar graphs were used to visualize the differences in relative expression levels between groups. Statistical analysis was performed by two-way ANOVA with two independent variables (genotypes and Mn treatment), followed by Tukey’s *post hoc* test for the open-field, rotarod, relative gene, and protein expression analyses. Mice for each group for the *in vivo* experiments were 15 to 20, and sample numbers of each group for *in vitro* cell culture experiments were 3 to 6. The data for *in vitro* experiments are representative of three independent experiments. A *p*-value of <0.05 was considered statistically significant.

## Data availability

All data that support this study are provided in the article.

## Conflicts of interest

The authors declare that they have no conflicts of interest with the contents of this article.
